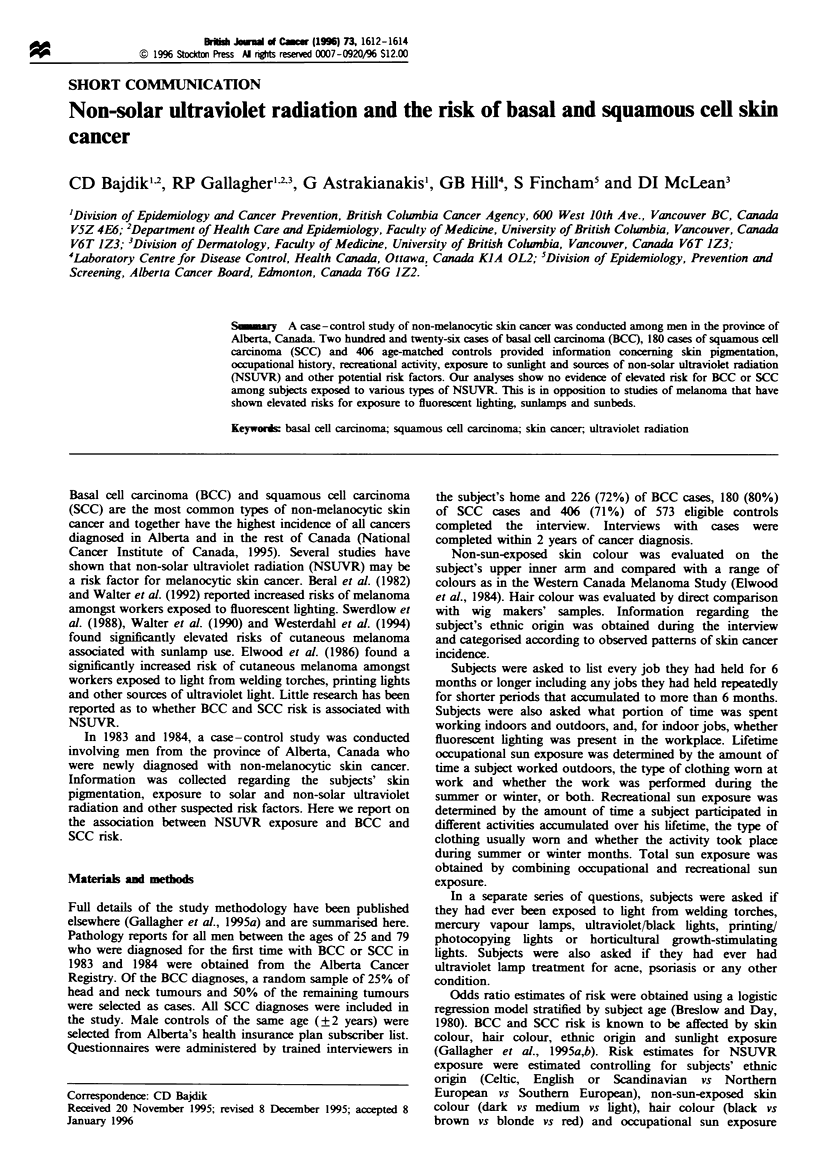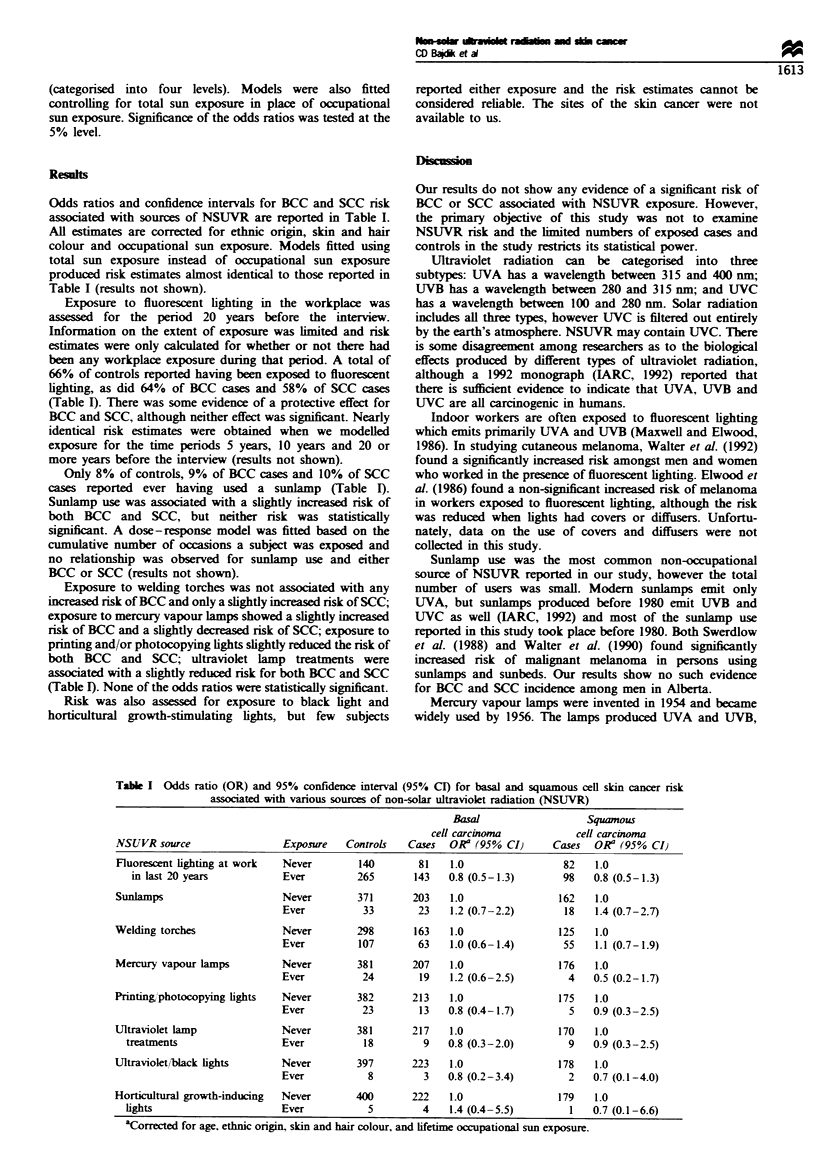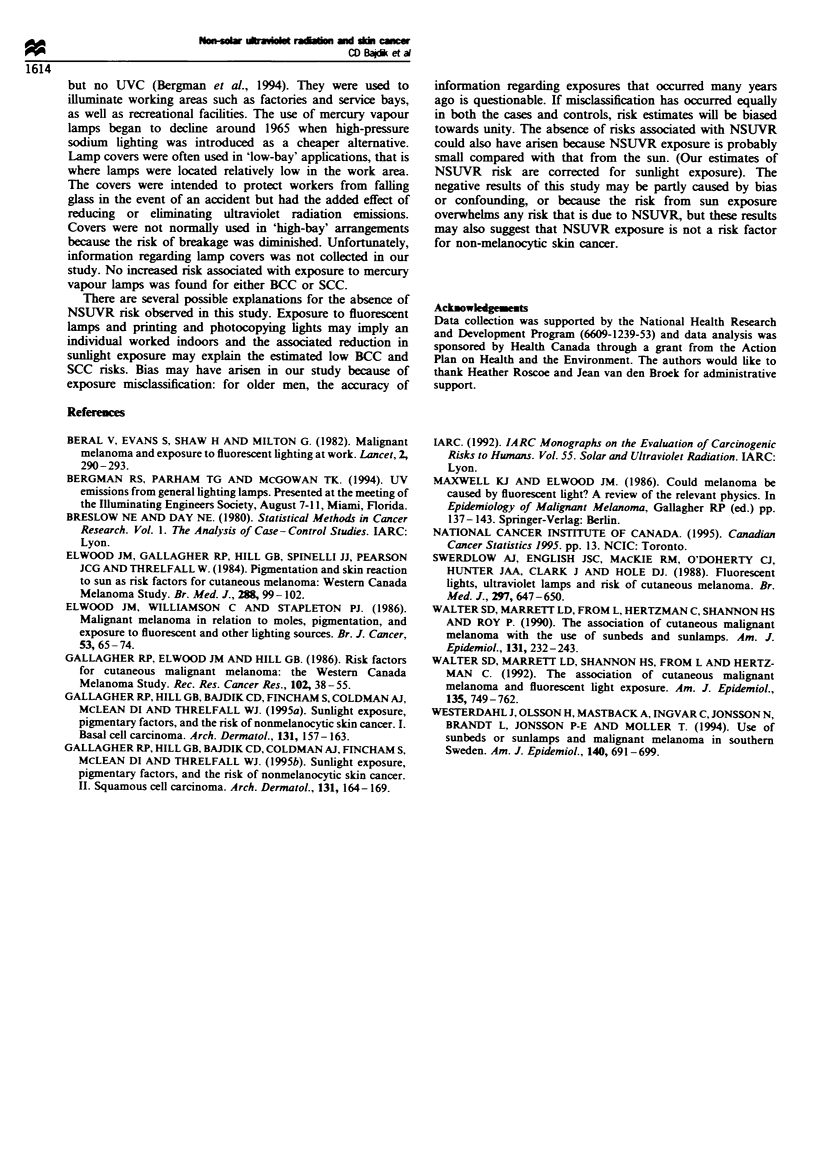# Non-solar ultraviolet radiation and the risk of basal and squamous cell skin cancer.

**DOI:** 10.1038/bjc.1996.303

**Published:** 1996-06

**Authors:** C. D. Bajdik, R. P. Gallagher, G. Astrakianakis, G. B. Hill, S. Fincham, D. I. McLean

**Affiliations:** Division of Epidemiology and Cancer Prevention, British Columbia Cancer Agency, Vancouver, Canada.

## Abstract

A case-control study of non-melanocytic skin cancer was conducted among men in the province of Alberta, Canada. Two hundred and twenty-six cases of basal cell carcinoma (BCC), 180 cases of squamous cell carcinoma (SCC) and 406 age-matched controls provided information concerning skin pigmentation, occupational history, recreational activity, exposure to sunlight and sources of non-solar ultraviolet radiation (NSUVR) and other potential risk factors. Our analyses show no evidence of elevated risk for BCC or SCC among subjects exposed to various types of NSUVR. This is in opposition to studies of melanoma that have shown elevated risks for exposure to fluorescent lighting, sunlamps and sunbeds.


British Journal of Cancer (1996) 73, 1612-1614

© 1996 Stockton Press All rights reserved 0007-0920/96 $12.00

SHORT COMMUNICATION

### Non-solar ultraviolet radiation and the risk of basal and squamous cell skin cancer

CD Bajdik[1,2], RP Gallagher[1,2,3], G Astrakianakis[1], GB Hill[4], S Fincham[5] and DI McLean[3]

[1]*Division of Epidemiology and Cancer Prevention, British Columbia Cancer Agency, 600 West 10th Ave., Vancouver BC, Canada V5Z 4E6;* [2]*Department of Health Care and Epidemiology, Faculty of Medicine, University of British Columbia, Vancouver, Canada V6T 1Z3;* [3]*Division of Dermatology, Faculty of Medicine, University of British Columbia, Vancouver, Canada V6T 1Z3;* [4]*Laboratory Centre for Disease Control, Health Canada, Ottawa, Canada K1A OL2;* [5]*Division of Epidemiology, Prevention and Screening, Alberta Cancer Board, Edmonton, Canada T6G 1Z2.*

**Summary** A case-control study of non-melanocytic skin cancer was conducted among men in the province of Alberta, Canada. Two hundred and twenty-six cases of basal cell carcinoma (BCC), 180 cases of squamous cell carcinoma (SCC) and 406 age-matched controls provided information concerning skin pigmentation, occupational history, recreational activity, exposure to sunlight and sources of non-solar ultraviolet radiation (NSUVR) and other potential risk factors. Our analyses show no evidence of elevated risk for BCC or SCC among subjects exposed to various types of NSUVR. This is in opposition to studies of melanoma that have shown elevated risks for exposure to fluorescent lighting, sunlamps and sunbeds.

**Keywords:** basal cell carcinoma; squamous cell carcinoma; skin cancer; ultraviolet radiation

Basal cell carcinoma (BCC) and squamous cell carcinoma (SCC) are the most common types of non-melanocytic skin cancer and together have the highest incidence of all cancers diagnosed in Alberta and in the rest of Canada (National Cancer Institute of Canada, 1995). Several studies have shown that non-solar ultraviolet radiation (NSUVR) may be a risk factor for melanocytic skin cancer. Beral *et al.* (1982) and Walter *et al.* (1992) reported increased risks of melanoma amongst workers exposed to fluorescent lighting. Swerdlow *et al.* (1988), Walter *et al.* (1990) and Westerdahl *et al.* (1994) found significantly elevated risks of cutaneous melanoma associated with sunlamp use. Elwood *et al.* (1986) found a significantly increased risk of cutaneous melanoma amongst workers exposed to light from welding torches, printing lights and other sources of ultraviolet light. Little research has been reported as to whether BCC and SCC risk is associated with NSUVR.

In 1983 and 1984, a case-control study was conducted involving men from the province of Alberta, Canada who were newly diagnosed with non-melanocytic skin cancer. Information was collected regarding the subjects' skin pigmentation, exposure to solar and non-solar ultraviolet radiation and other suspected risk factors. Here we report on the association between NSUVR exposure and BCC and SCC risk.

### Materials and methods

Full details of the study methodology have been published elsewhere (Gallagher *et al.*, 1995*a*) and are summarised here. Pathology reports for all men between the ages of 25 and 79 who were diagnosed for the first time with BCC or SCC in 1983 and 1984 were obtained from the Alberta Cancer Registry. Of the BCC diagnoses, a random sample of 25% of head and neck tumours and 50% of the remaining tumours were selected as cases. All SCC diagnoses were included in the study. Male controls of the same age ($\pm 2$ years) were selected from Alberta's health insurance plan subscriber list. Questionnaires were administered by trained interviewers in the subject's home and 226 (72%) of BCC cases, 180 (80%) of SCC cases and 406 (71%) of 573 eligible controls completed the interview. Interviews with cases were completed within 2 years of cancer diagnosis.

Non-sun-exposed skin colour was evaluated on the subject's upper inner arm and compared with a range of colours as in the Western Canada Melanoma Study (Elwood *et al.*, 1984). Hair colour was evaluated by direct comparison with wig makers' samples. Information regarding the subject's ethnic origin was obtained during the interview and categorised according to observed patterns of skin cancer incidence.

Subjects were asked to list every job they had held for 6 months or longer including any jobs they had held repeatedly for shorter periods that accumulated to more than 6 months. Subjects were also asked what portion of time was spent working indoors and outdoors, and, for indoor jobs, whether fluorescent lighting was present in the workplace. Lifetime occupational sun exposure was determined by the amount of time a subject worked outdoors, the type of clothing worn at work and whether the work was performed during the summer or winter, or both. Recreational sun exposure was determined by the amount of time a subject participated in different activities accumulated over his lifetime, the type of clothing usually worn and whether the activity took place during summer or winter months. Total sun exposure was obtained by combining occupational and recreational sun exposure.

In a separate series of questions, subjects were asked if they had ever been exposed to light from welding torches, mercury vapour lamps, ultraviolet/black lights, printing/photocopying lights or horticultural growth-stimulating lights. Subjects were also asked if they had ever had ultraviolet lamp treatment for acne, psoriasis or any other condition.

Odds ratio estimates of risk were obtained using a logistic regression model stratified by subject age (Breslow and Day, 1980). BCC and SCC risk is known to be affected by skin colour, hair colour, ethnic origin and sunlight exposure (Gallagher *et al.*, 1995*a*,*b*). Risk estimates for NSUVR exposure were estimated controlling for subjects' ethnic origin (Celtic, English or Scandinavian *vs* Northern European *vs* Southern European), non-sun-exposed skin colour (dark *vs* medium *vs* light), hair colour (black *vs* brown *vs* blonde *vs* red) and occupational sun exposure

Correspondence: CD Bajdik
Received 20 November 1995; revised 8 December 1995; accepted 8 January 1996

(categorised into four levels). Models were also fitted controlling for total sun exposure in place of occupational sun exposure. Significance of the odds ratios was tested at the 5% level.

### Results

Odds ratios and confidence intervals for BCC and SCC risk associated with sources of NSUVR are reported in Table I. All estimates are corrected for ethnic origin, skin and hair colour and occupational sun exposure. Models fitted using total sun exposure instead of occupational sun exposure produced risk estimates almost identical to those reported in Table I (results not shown).

Exposure to fluorescent lighting in the workplace was assessed for the period 20 years before the interview. Information on the extent of exposure was limited and risk estimates were only calculated for whether or not there had been any workplace exposure during that period. A total of 66% of controls reported having been exposed to fluorescent lighting, as did 64% of BCC cases and 58% of SCC cases (Table I). There was some evidence of a protective effect for BCC and SCC, although neither effect was significant. Nearly identical risk estimates were obtained when we modelled exposure for the time periods 5 years, 10 years and 20 or more years before the interview (results not shown).

Only 8% of controls, 9% of BCC cases and 10% of SCC cases reported ever having used a sunlamp (Table I). Sunlamp use was associated with a slightly increased risk of both BCC and SCC, but neither risk was statistically significant. A dose–response model was fitted based on the cumulative number of occasions a subject was exposed and no relationship was observed for sunlamp use and either BCC or SCC (results not shown).

Exposure to welding torches was not associated with any increased risk of BCC and only a slightly increased risk of SCC; exposure to mercury vapour lamps showed a slightly increased risk of BCC and a slightly decreased risk of SCC; exposure to printing and/or photocopying lights slightly reduced the risk of both BCC and SCC; ultraviolet lamp treatments were associated with a slightly reduced risk for both BCC and SCC (Table I). None of the odds ratios were statistically significant.

Risk was also assessed for exposure to black light and horticultural growth-stimulating lights, but few subjects reported either exposure and the risk estimates cannot be considered reliable. The sites of the skin cancer were not available to us.

### Discussion

Our results do not show any evidence of a significant risk of BCC or SCC associated with NSUVR exposure. However, the primary objective of this study was not to examine NSUVR risk and the limited numbers of exposed cases and controls in the study restricts its statistical power.

Ultraviolet radiation can be categorised into three subtypes: UVA has a wavelength between 315 and 400 nm; UVB has a wavelength between 280 and 315 nm; and UVC has a wavelength between 100 and 280 nm. Solar radiation includes all three types, however UVC is filtered out entirely by the earth's atmosphere. NSUVR may contain UVC. There is some disagreement among researchers as to the biological effects produced by different types of ultraviolet radiation, although a 1992 monograph (IARC, 1992) reported that there is sufficient evidence to indicate that UVA, UVB and UVC are all carcinogenic in humans.

Indoor workers are often exposed to fluorescent lighting which emits primarily UVA and UVB (Maxwell and Elwood, 1986). In studying cutaneous melanoma, Walter *et al.* (1992) found a significantly increased risk amongst men and women who worked in the presence of fluorescent lighting. Elwood *et al.* (1986) found a non-significant increased risk of melanoma in workers exposed to fluorescent lighting, although the risk was reduced when lights had covers or diffusers. Unfortunately, data on the use of covers and diffusers were not collected in this study.

Sunlamp use was the most common non-occupational source of NSUVR reported in our study, however the total number of users was small. Modern sunlamps emit only UVA, but sunlamps produced before 1980 emit UVB and UVC as well (IARC, 1992) and most of the sunlamp use reported in this study took place before 1980. Both Swerdlow *et al.* (1988) and Walter *et al.* (1990) found significantly increased risk of malignant melanoma in persons using sunlamps and sunbeds. Our results show no such evidence for BCC and SCC incidence among men in Alberta.

Mercury vapour lamps were invented in 1954 and became widely used by 1956. The lamps produced UVA and UVB,

**Table I** Odds ratio (OR) and 95% confidence interval (95% CI) for basal and squamous cell skin cancer risk associated with various sources of non-solar ultraviolet radiation (NSUVR)

| | | | *Basal cell carcinoma* | | *Squamous cell carcinoma* | |
|---|---|---|---|---|---|---|
| *NSUVR source* | *Exposure* | *Controls* | *Cases* | *OR[a] (95% CI)* | *Cases* | *OR[a] (95% CI)* |
| Fluorescent lighting at work in last 20 years | Never | 140 | 81 | 1.0 | 82 | 1.0 |
| | Ever | 265 | 143 | 0.8 (0.5–1.3) | 98 | 0.8 (0.5–1.3) |
| Sunlamps | Never | 371 | 203 | 1.0 | 162 | 1.0 |
| | Ever | 33 | 23 | 1.2 (0.7–2.2) | 18 | 1.4 (0.7–2.7) |
| Welding torches | Never | 298 | 163 | 1.0 | 125 | 1.0 |
| | Ever | 107 | 63 | 1.0 (0.6–1.4) | 55 | 1.1 (0.7–1.9) |
| Mercury vapour lamps | Never | 381 | 207 | 1.0 | 176 | 1.0 |
| | Ever | 24 | 19 | 1.2 (0.6–2.5) | 4 | 0.5 (0.2–1.7) |
| Printing/photocopying lights | Never | 382 | 213 | 1.0 | 175 | 1.0 |
| | Ever | 23 | 13 | 0.8 (0.4–1.7) | 5 | 0.9 (0.3–2.5) |
| Ultraviolet lamp treatments | Never | 381 | 217 | 1.0 | 170 | 1.0 |
| | Ever | 18 | 9 | 0.8 (0.3–2.0) | 9 | 0.9 (0.3–2.5) |
| Ultraviolet/black lights | Never | 397 | 223 | 1.0 | 178 | 1.0 |
| | Ever | 8 | 3 | 0.8 (0.2–3.4) | 2 | 0.7 (0.1–4.0) |
| Horticultural growth-inducing lights | Never | 400 | 222 | 1.0 | 179 | 1.0 |
| | Ever | 5 | 4 | 1.4 (0.4–5.5) | 1 | 0.7 (0.1–6.6) |

[a]Corrected for age, ethnic origin, skin and hair colour, and lifetime occupational sun exposure.

but no UVC (Bergman *et al.*, 1994). They were used to illuminate working areas such as factories and service bays, as well as recreational facilities. The use of mercury vapour lamps began to decline around 1965 when high-pressure sodium lighting was introduced as a cheaper alternative. Lamp covers were often used in 'low-bay' applications, that is where lamps were located relatively low in the work area. The covers were intended to protect workers from falling glass in the event of an accident but had the added effect of reducing or eliminating ultraviolet radiation emissions. Covers were not normally used in 'high-bay' arrangements because the risk of breakage was diminished. Unfortunately, information regarding lamp covers was not collected in our study. No increased risk associated with exposure to mercury vapour lamps was found for either BCC or SCC.

There are several possible explanations for the absence of NSUVR risk observed in this study. Exposure to fluorescent lamps and printing and photocopying lights may imply an individual worked indoors and the associated reduction in sunlight exposure may explain the estimated low BCC and SCC risks. Bias may have arisen in our study because of exposure misclassification: for older men, the accuracy of information regarding exposures that occurred many years ago is questionable. If misclassification has occurred equally in both the cases and controls, risk estimates will be biased towards unity. The absence of risks associated with NSUVR could also have arisen because NSUVR exposure is probably small compared with that from the sun. (Our estimates of NSUVR risk are corrected for sunlight exposure). The negative results of this study may be partly caused by bias or confounding, or because the risk from sun exposure overwhelms any risk that is due to NSUVR, but these results may also suggest that NSUVR exposure is not a risk factor for non-melanocytic skin cancer.

**Acknowledgements**

Data collection was supported by the National Health Research and Development Program (6609-1239-53) and data analysis was sponsored by Health Canada through a grant from the Action Plan on Health and the Environment. The authors would like to thank Heather Roscoe and Jean van den Broek for administrative support.

### References

BERAL V, EVANS S, SHAW H AND MILTON G. (1982). Malignant melanoma and exposure to fluorescent lighting at work. *Lancet*, **2**, 290-293.

BERGMAN RS, PARHAM TG AND MCGOWAN TK. (1994). UV emissions from general lighting lamps. Presented at the meeting of the Illuminating Engineers Society, August 7-11, Miami, Florida.

BRESLOW NE AND DAY NE. (1980). *Statistical Methods in Cancer Research. Vol. 1. The Analysis of Case-Control Studies*. IARC: Lyon.

ELWOOD JM, GALLAGHER RP, HILL GB, SPINELLI JJ, PEARSON JCG AND THRELFALL W. (1984). Pigmentation and skin reaction to sun as risk factors for cutaneous melanoma: Western Canada Melanoma Study. *Br. Med. J.*, **288**, 99-102.

ELWOOD JM, WILLIAMSON C AND STAPLETON PJ. (1986). Malignant melanoma in relation to moles, pigmentation, and exposure to fluorescent and other lighting sources. *Br. J. Cancer*, **53**, 65-74.

GALLAGHER RP, ELWOOD JM AND HILL GB. (1986). Risk factors for cutaneous malignant melanoma: the Western Canada Melanoma Study. *Rec. Res. Cancer Res.*, **102**, 38-55.

GALLAGHER RP, HILL GB, BAJDIK CD, FINCHAM S, COLDMAN AJ, MCLEAN DI AND THRELFALL WJ. (1995*a*). Sunlight exposure, pigmentary factors, and the risk of nonmelanocytic skin cancer. I. Basal cell carcinoma. *Arch. Dermatol.*, **131**, 157-163.

GALLAGHER RP, HILL GB, BAJDIK CD, COLDMAN AJ, FINCHAM S, MCLEAN DI AND THRELFALL WJ. (1995*b*). Sunlight exposure, pigmentary factors, and the risk of nonmelanocytic skin cancer. II. Squamous cell carcinoma. *Arch. Dermatol.*, **131**, 164-169.

IARC. (1992). *IARC Monographs on the Evaluation of Carcinogenic Risks to Humans. Vol. 55. Solar and Ultraviolet Radiation*. IARC: Lyon.

MAXWELL KJ AND ELWOOD JM. (1986). Could melanoma be caused by fluorescent light? A review of the relevant physics. In *Epidemiology of Malignant Melanoma*, Gallagher RP (ed.) pp. 137-143. Springer-Verlag: Berlin.

NATIONAL CANCER INSTITUTE OF CANADA. (1995). *Canadian Cancer Statistics 1995*. pp. 13. NCIC: Toronto.

SWERDLOW AJ, ENGLISH JSC, MACKIE RM, O'DOHERTY CJ, HUNTER JAA, CLARK J AND HOLE DJ. (1988). Fluorescent lights, ultraviolet lamps and risk of cutaneous melanoma. *Br. Med. J.*, **297**, 647-650.

WALTER SD, MARRETT LD, FROM L, HERTZMAN C, SHANNON HS AND ROY P. (1990). The association of cutaneous malignant melanoma with the use of sunbeds and sunlamps. *Am. J. Epidemiol.*, **131**, 232-243.

WALTER SD, MARRETT LD, SHANNON HS, FROM L AND HERTZMAN C. (1992). The association of cutaneous malignant melanoma and fluorescent light exposure. *Am. J. Epidemiol.*, **135**, 749-762.

WESTERDAHL J, OLSSON H, MASTBACK A, INGVAR C, JONSSON N, BRANDT L, JONSSON P-E AND MOLLER T. (1994). Use of sunbeds or sunlamps and malignant melanoma in southern Sweden. *Am. J. Epidemiol.*, **140**, 691-699.